# Identification and validation of a novel mitochondrion-related gene signature for diagnosis and immune infiltration in sepsis

**DOI:** 10.3389/fimmu.2023.1196306

**Published:** 2023-06-15

**Authors:** Shuai Hao, Miao Huang, Xiaofan Xu, Xulin Wang, Yuqing Song, Wendi Jiang, Liqun Huo, Jun Gu

**Affiliations:** ^1^ Department of General Surgery, Jinling Hospital, Medical School of Nanjing University, Nanjing, China; ^2^ Nursing School, Chongqing Medical University, Chongqing, China

**Keywords:** sepsis, immune infiltration, mitochondrion, diagnostic biomarkers, bioinformatics

## Abstract

**Background:**

Owing to the complex pathophysiological features and heterogeneity of sepsis, current diagnostic methods are not sufficiently precise or timely, causing a delay in treatment. It has been suggested that mitochondrial dysfunction plays a critical role in sepsis. However, the role and mechanism of mitochondria-related genes in the diagnostic and immune microenvironment of sepsis have not been sufficiently investigated.

**Methods:**

Mitochondria-related differentially expressed genes (DEGs) were identified between human sepsis and normal samples from GSE65682 dataset. Least absolute shrinkage and selection operator (LASSO) regression and the Support Vector Machine (SVM) analyses were carried out to locate potential diagnostic biomarkers. Gene ontology and gene set enrichment analyses were conducted to identify the key signaling pathways associated with these biomarker genes. Furthermore, correlation of these genes with the proportion of infiltrating immune cells was estimated using CIBERSORT. The expression and diagnostic value of the diagnostic genes were evaluated using GSE9960 and GSE134347 datasets and septic patients. Furthermore, we established an *in vitro* sepsis model using lipopolysaccharide (1 µg/mL)-stimulated CP-M191 cells. Mitochondrial morphology and function were evaluated in PBMCs from septic patients and CP-M191 cells, respectively.

**Results:**

In this study, 647 mitochondrion-related DEGs were obtained. Machine learning confirmed six critical mitochondrion-related DEGs, including *PID1*, *CS*, *CYP1B1*, *FLVCR1*, *IFIT2*, and *MAPK14*. We then developed a diagnostic model using the six genes, and receiver operating characteristic (ROC) curves indicated that the novel diagnostic model based on the above six critical genes screened sepsis samples from normal samples with area under the curve (AUC) = 1.000, which was further demonstrated in the GSE9960 and GSE134347 datasets and our cohort. Importantly, we also found that the expression of these genes was associated with different kinds of immune cells. In addition, mitochondrial dysfunction was mainly manifested by the promotion of mitochondrial fragmentation (p<0.05), impaired mitochondrial respiration (p<0.05), decreased mitochondrial membrane potential (p<0.05), and increased reactive oxygen species (ROS) generation (p<0.05) in human sepsis and LPS-simulated *in vitro* sepsis models.

**Conclusion:**

We constructed a novel diagnostic model containing six MRGs, which has the potential to be an innovative tool for the early diagnosis of sepsis.

## 1 Introduction

Sepsis is defined as organ dysfunction syndrome caused by an uncontrolled inflammatory response to infection (1, 2). It is one of the leading causes of death and is responsible for 30% of all fatal cases that occur in hospitalized patients (3, 4). The septic response is an extremely complicated chain of events that includes humoral and cellular reactions, inflammatory and anti-inflammatory processes, and circulatory abnormalities (5). Even though mortality and morbidity rates are high, no sepsis-specific treatments are currently available for clinical use (6). The highly variable nature of the signs and symptoms of sepsis makes it difficult to diagnose and determine its severity when it has already developed. Given this, making an early diagnosis and categorizing the severity of sepsis is critical because timely implementation of targeted interventions improves outcomes (7).

Over the past few decades, researchers have studied the different phases of the inflammatory response during sepsis and septic shock. Dysregulated host response or immune dysfunction is related to defective natural killer (NK) cell activity, defective antigen presentation, neutrophil abnormalities, decreased immunoglobulin levels, complement consumption, hypercytokinemia, and defective bacterial removal (8, 9). A multitude of related biomarkers has been proposed to aid the diagnosis of sepsis, such as procalcitonin, C-reactive protein, and lactate (10, 11). However, these biomarkers are limited by their sensitivity and specificity and do not accurately assess the development of sepsis. Therefore, it is crucial to explore the pathogenesis of sepsis in depth and screen for new targets.

Recent studies have shown that cell death is relatively uncommon in sepsis, which suggests that processes other than cell death are responsible for mortality (12, 13). Moreover, accumulating evidence supports the hypothesis that the inability of the cell to use oxygen as a fuel source may play a significant role in the pathogenesis of sepsis (14, 15). Because mitochondrial O_2_ consumption accounts for 90% of the body’s total O_2_ consumption, impaired O_2_ utilization and dysfunctional mitochondria may be responsible for the distinctive features of sepsis (16, 17). In addition, septic patients produce an abnormally high amount of oxidants. Consequently, these oxidants might be the source of the abnormalities described above, which ultimately results in an increased mortality rate.

Mitochondria are specialized intracellular structures enclosed by a double-layer membrane and are the hub for cellular metabolism, participating in a series of important cellular processes, including oxidative phosphorylation, the tricarboxylic acid cycle, maintenance of intracellular calcium homeostasis, and production and maintenance of reactive oxygen species (ROS) (18, 19). During sepsis development, the mitochondria undergo morphological and functional damage in many ways. Liang et al. found that the mitochondrial morphology of cardiomyocytes in an animal model of sepsis was irregular, with significant swelling and vacuolar degeneration, and evident mitochondrial crest rupture was noted in the swelling mitochondria (20). Zhang et al. found similar results. In addition to abnormal mitochondrial morphology, sepsis impairs mitochondrial function, resulting in a significant decrease in mitochondrial membrane potential (21). However, the specific mechanisms still require further investigation. Gene expression profiles obtained from septic patients provide a wealth of information that could mitigate the effects of selection bias and illustrate a wide variety of biological responses (22, 23). The understanding of sepsis is supported by genes and pathways that are up- or downregulated in relation to the condition. Thus, we were curious whether mitochondrion-related genes could be utilized as novel diagnostic factors for sepsis.

In recent years, an increasing number of studies have used machine-learning algorithms to screen prospective diagnostic genes in a variety of disorders based on high-throughput sequencing (24–26). Using machine learning techniques, the primary objective of this study was to locate important diagnostic mitochondrion-related genes. In addition, we further investigated their relationship to immunological infiltration. Furthermore, using Transmission electron microscopy (TEM) analysis, mitochondrial respiration measurements, mitochondrial membrane potential, and mitochondrial ROS production analyses, we observed mitochondrial morphology and function changes in peripheral blood mononuclear cells (PBMCs) derived from septic patients as well as in CP-M191 cells.

## 2 Materials and methods

### 2.1 Data collection and preprocessing

Public gene expression matrices (GSE65682, GSE9960, GSE134347) were obtained from the Gene Expression Omnibus (GEO) database (https://www.ncbi.nlm.nih.gov/geo/). The GSE65682 dataset included 802 samples, which comprised 40 healthy controls and 760 septic patients (**Table S1**). Gene expression profiles of PBMC using array technology were included in the GSE9960 dataset. These profiles were obtained from 54 adult septic patients and 16 healthy controls. The primary data accession number for the HTA2.0 microarray data acquired from GEO was GSE134347. This dataset included 82 healthy individuals and 156 septic patients. The lumi package in R was used to process the raw data included within these three datasets. The 1513 mitochondrion-related genes were searched from the Gene Set Enrichment Analysis (GSEA), Gene Cards, and UniProt databases (27) and are shown in **Table S2**.

### 2.2 Differential expression analysis

We accessed the GSE65682 database and collected the expression data of all 1513 mitochondria-related genes (MRGs) from normal samples as well as samples from septic patients. Following that, the Wilcoxon Rank-Sum Test was carried out in R in order to identify the genes associated with mitochondria that exhibited distinct levels of expression in the two distinct samples. Genes that had a p-value of less than 0.05 were regarded as significant.

### 2.3 Function enrichment analysis of differentially expressed genes

Gene Ontology (GO) analysis and Kyoto Encyclopedia of Genes and Genomes (KEGG) pathway enrichment analyses were carried out using clusterProfiler (28) software to determine the potential functions and pathways of differentially expressed MRGs (DEMRGs). The visualization module found in clusterProfiler was used to display the results of the analysis. The cutoff value was set at P < 0.05. The “clusterProfiler” and DOSE packages in R were utilized in order to conduct disease ontology (DO) enrichment analyses on DEMRGs. GSEA is a computational method used to determine whether a predefined set of genes exhibits significant differences between two biological states and is commonly used to estimate changes in pathway and biological process activity in samples from expression datasets.

For the reference gene set, we used the “c2.cp.kegg.v7.0. symbols.gmt” file from the Molecular Signatures Database (MSigDB) (29). If the value of P < 0.05, and the false discovery rate < 0.025, then a gene collection was considered to be significantly enriched.

### 2.4 Construction of the LASSO model and the SVM-RFE feature selection process

Diagnostic MRGs of sepsis were categorized using least absolute shrinkage and selection operator (LASSO) regression and the Support Vector Machine (SVM) algorithm. The LASSO algorithm was performed using the “glmnet (4.1.7)” package. The response type was configured to be binomial, and the alpha was set to 1. In addition, SVM is a surveillant machine learning approach to support vectors. It discovers the best variables by eliminating the feature vectors generated by the SVM. The SVM classifier found in the R package e1071 (1.7.13) was used to classify the chosen biomarkers in the sepsis diagnosis process. The k-fold cross validation was set to a value of five, and the parameter of halving above was determined to be 100.

### 2.5 ceRNA

Competing endogenous RNA (ceRNA) is a role element that can compete to bind to RNA. A ceRNA regulatory network refers to the entire regulatory network involving ceRNAs, which usually consists of mRNA, miRNAs, and lncRNAs. We constructed the mRNA–miRNA–lncRNA regulatory network using the starBase and miRanda databases, which was subsequently visualized using Cytoscape.

### 2.6 Immune cell infiltration

A bioinformatics tool known as CIBERSORT (https://cibersortx.stanford.edu/) was utilized to compute immune cell infiltration in sepsis. This was performed to quantify the relative proportions of infiltrating immune cells based on the gene expression profiles observed in sepsis. A reference set consisting of 22 distinct immune cell subtypes (LM22) and 1000 different permutations was utilized to estimate the potential number of immune cells. The “corrplot” R package was used to do correlation analysis as well as visualization of 22 different types of immune cells. To visualize the variations in immune cell infiltration between the sepsis and control samples, violin plots were generated using the “vioplot” R package. Correlation analyses were performed to analyze the relationships between the expression of MRGs and immune cells.

### 2.7 Patients and samples

A total of 15 septic patients and 15 healthy volunteers at The Second Affiliated Hospital of Chongqing Medical University between September 2021 and July 2022 were included in the present study, and their blood samples were provided. The participants or their legal representatives were asked to provide written informed permission for the study. The study was approved by the Medical Ethics Committee of The Second Affiliated Hospital of Chongqing Medical University (ID:2022-190). The Third International Consensus Definitions for Sepsis and Septic Shock (Sepsis-3) were used to diagnose sepsis (30).

Blood samples were collected by venipuncture, and PBMCs were separated using Ficoll-Paque density gradient centrifugation as per the manufacturer’s instructions (31). PBMC (1 × 10^5^ cells/well) were cultured in complete RPMI-1640 media as usual and prepared for subsequent experiments.

### 2.8 Model preparation

CP-M191 cells were cultured in Dulbecco’s modified eagle medium (DMEM) supplemented with 10% fetal bovine serum, maintained at 37°C, and cultured in a humidified environment of 5% CO_2_/95% air. When the cells reached a confluence of over 70% in the culture medium, they were randomly treated with DMEM (CON group) or 1 µg/mL LPS (LPS group) for 24 h. After treatment, the cells were collected for relevant experiments.

### 2.9 Quantitative RT-PCR

Total RNA was extracted from each sample using TRIzol reagent (Invitrogen, Carlsbad, CA, USA). Using a PrimeScript™ RT kit (Takara, Japan), RNA was reverse transcribed into cDNA to detect relative mRNA levels using qPCR (Bio-Rad). Relative gene expression levels were calculated using the 2-ΔΔct method. GAPDH was used as an internal control. Primers used in these experiments are listed in **Table S3**. All experiments were repeated three times.

### 2.10 Transmission electron microscopy

PBMCs were fixed in 2.5% (w/v) glutaraldehyde for 24 h, washed with PBS three times, stained with 1% osmium tetroxide, and then dehydrated in a graded series of ethanol (65%, 70%, 75%, 80%, and 95% for 10 min each). Subsequently, the samples were incubated with tert-butoxide for 10 min and then dried with CO_2_ (carbon dioxide), stained with uranyl acetate, and coated with gold (Au) using an ion sputter coater. Finally, the samples were viewed and imaged using a transmission electron microscope (H-7500, Hitachi Company, Japan) (32).

### 2.11 Mitochondrial morphology

PBMCs were seeded on confocal culture plates at a density of 1 × 10^5^ cells/well and cultured for approximately 2 days for confocal imaging. Mitochondrial morphology was observed as previously described (33). MitoTracker (Deep Red, 100 nM) was added, and the cells were incubated for 30 min. Subsequently, the mitochondria were observed using an inverted confocal microscope (Leica TCS SP5; Leica Microsystems) with a 60 × 1:3 NA oil immersion objective. Red fluorescence was excited using a 633 nm laser, and the emission spectra were obtained at 655–670 nm. Image-Pro Plus software was used to measure and compute the mitochondrial length and aspect ratios.

### 2.12 Measurement of intracellular ROS

Intracellular ROS levels were measured as previously described (34). The ROS assay working solution was added to the cells, which were then incubated for 30 min. The cells were imaged using a Leica TCS SP5. Green fluorescence was excited with a 488 nm laser, and emission spectra were obtained at 501–563 nm. Images were analyzed using Image-Pro Plus software.

### 2.13 Mitochondrial membrane potential (△Ψm) assay

Mitochondrial membrane potential was measured as previously described (35). JC-1 staining working solution was added to the cells, which were then incubated for 30 min. The cells were imaged using a Leica TCS SP5. The monomer was excited with a 488-nm laser, and emission spectra were obtained at 501–563 nm. Aggregate fluorescence was excited using a 633-nm laser, and emission spectra were obtained at 558–617 nm. Images were analyzed using Image-Pro Plus software.

### 2.14 Mitochondrial oxygen consumption rate

The oxygen consumption rate (OCR) was measured using a 24-well XFe plate (Seahorse, Agilent Cell Analysis Technology, USA). When cells reached 70% confluence (3-4 × 10^4^/well), cells were treated with LPS for 12 h. Before detection, the basic assay medium contained 2.5 μM glucose and 2 mM glutamine was used to culture cells for 50 min, after which 2 μM oligomycin, 1 μM FCCP, and 0.5 μM rotenone/antimycin A were sequentially added. The OCR was measured using an extracellular flux analyzer under mitochondrial stress test conditions.

### 2.15 Statistical analysis

Statistical analyses were performed using R software (https://www.r-project.org/, v4.0.1). For between-group comparisons of continuous variables, an independent t-test was used to compare normally distributed variables, and a Wilcoxon rank-sum test was used to compare non-normally distributed variables. A receiver operating characteristic (ROC) curve was plotted to predict binary categorical variables using the pROC package. All p-values were two-sided, and statistical significance was set at p < 0.05.

## 3 Results

### 3.1 Identification of DEGs in sepsis

**Figure 1:**
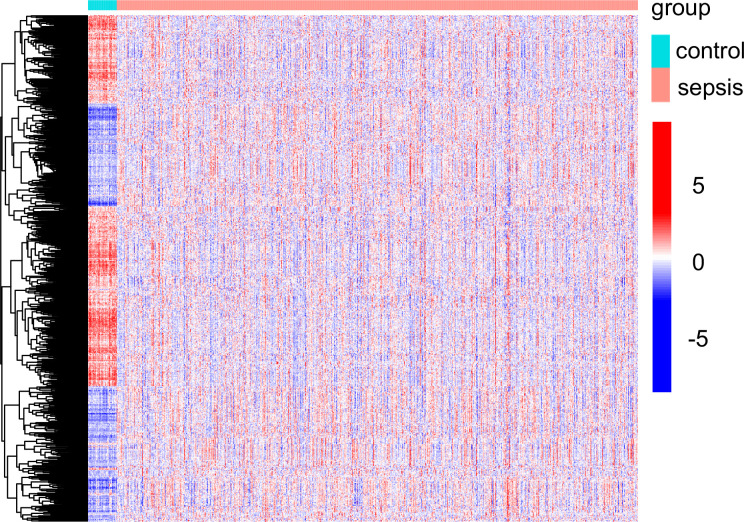
Mitochondria-related DEGs between sepsis samples and normal samples from GSE65682 datasets.

In this study, retrospective data analysis was conducted on 760 sepsis samples and 42 control samples obtained from the GSE65682 datasets. A total of 647 DEGs were discovered that were associated with mitochondria. Among them, 294 genes were significantly upregulated, while 353 genes were significantly downregulated (**Figure 1**). Workflow of the study was shown in **Figure S1**.

### 3.2 Functional enrichment analysis of DEGs

**Figure 2:**
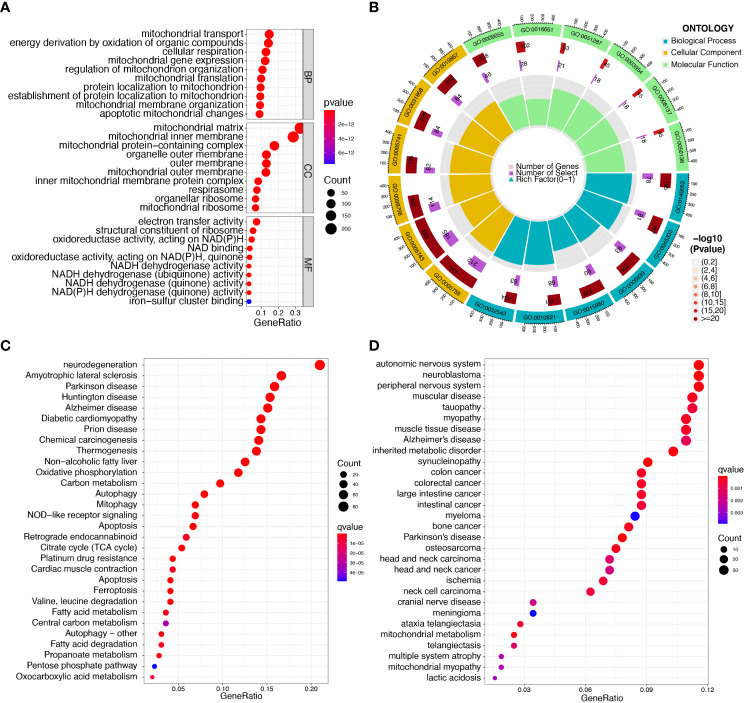
Functional enrichment analysis of DEGs in sepsis samples. **(A, B)** The most significantly enriched GO terms of DEGs. **(C)** KEGG pathway enrichment analysis of DEGs. **(D)** Disease ontology enrichment analysis of DEGs.

Enrichment analyses of GO and KEGG were performed with the help of the ClusterProfiler software package to investigate the potential biological function of mitochondria-related DEGs. The results of GO analyses indicated that the DEGs were mainly associated with mitochondrial transport, energy derivation by oxidation of organic compounds, cellular respiration, mitochondrial gene expression, mitochondrial matrix, mitochondrial inner membrane, electron transfer activity, and structural constituents of the ribosome (**Figures 2A, B**, **Table S4**). We then performed KEGG assays and found that the DEGs were mainly enriched in pathways of neurodegeneration-multiple diseases, amyotrophic lateral sclerosis, Parkinson’s disease, Huntington’s disease, Alzheimer’s disease, and diabetic cardiomyopathy (**Figure 2C**, **Table S5**). Finally, DO assays indicated that the DEGs were mainly related to autonomic nervous system neoplasm, neuroblastoma, peripheral nervous system neoplasm, muscular disease, tauopathy, myopathy, and muscle tissue disease (**Figure 2D**, **Table S6**).

### 3.3 Identification and construction of a diagnostic model for sepsis

**Figure 3:**
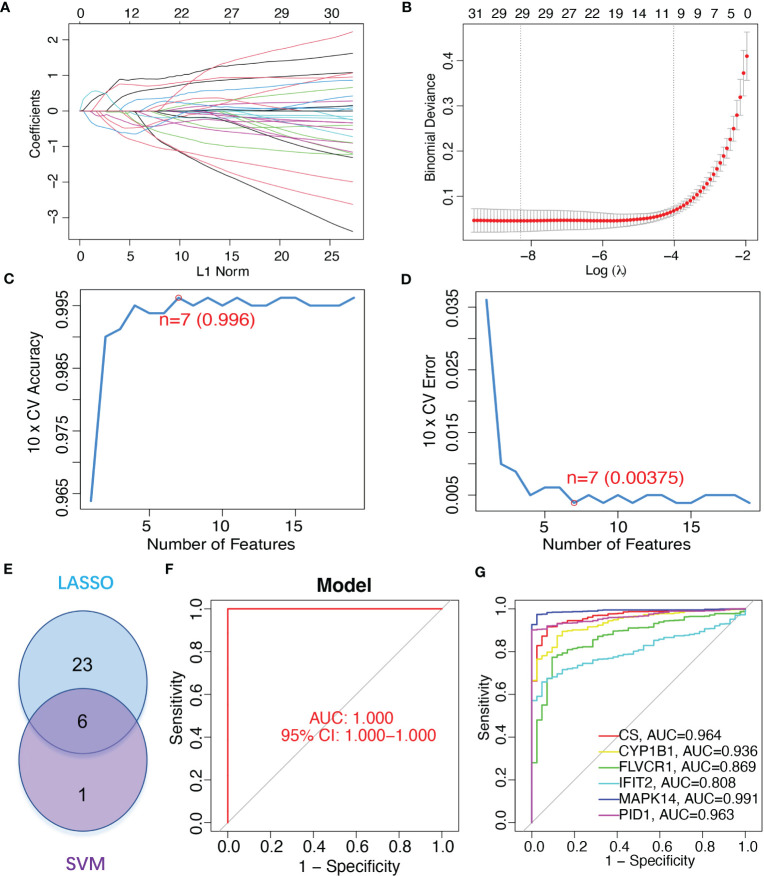
Gene signature of six mitochondrion-related genes was identified as a diagnostic model for sepsis. **(A, B)** LASSO methods. **(C, D)** SVM-RFE algorithm to identify the optimal combination of feature genes. **(E)** Critical genes from the LASSO and SVM-RFE methods. **(F)** AUC of sepsis using ROC assays. **(G)** ROC assays for the six critical genes.

To identify mitochondrion-related biomarkers of sepsis, two distinct machine-learning algorithms were applied. Using the LASSO algorithm, 29 variables were identified as diagnostic biomarkers for sepsis (**Figures 3A, B**). In addition, the SVM-RFE algorithm was used to narrow down the features of mitochondria-related DEGs to a subset of seven variables (**Figures 3C, D**). The final choice was made based on the six shared genes of these two algorithms: *CS*, *CYP1B1*, *FLVCR1*, *IFIT2*, *MAPK14*, and *PID1* (**Figure 3E**). We created a logistic regression model using the R package glm based on the aforementioned six marker genes, and ROC assays showed that the six gene-based logistic regression model distinguished normal and sepsis samples with an area under the curve (AUC) value of 1.000 (**Figure 3F**). In addition, ROC curves were constructed for the six marker genes in order to shed light on the individual genes’ capabilities in terms of identifying sepsis from normal samples. The AUC was higher than 0.8 for every gene, as shown in **Figure 3G**. Based on the information shown above, it appears that the novel diagnostic model provided a higher level of accuracy and specificity than the individual marker genes in distinguishing sepsis samples from normal samples.

### 3.4 Functional assays of the six diagnostic genes using GSEA pathway analysis

**Figure 4:**
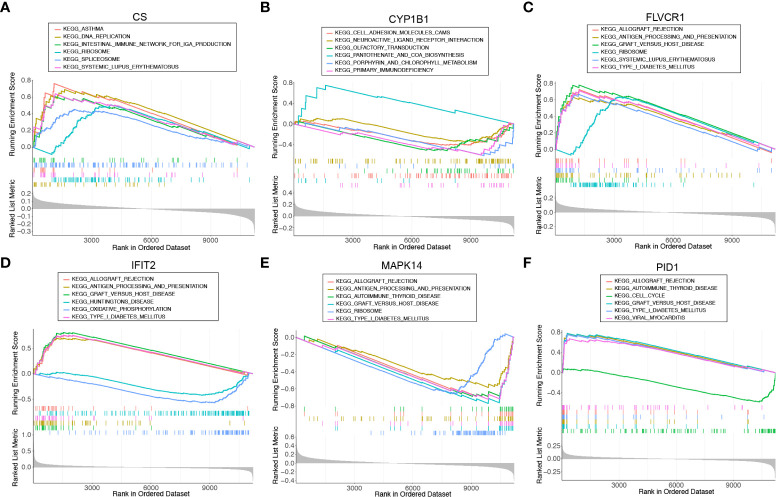
Single-gene GSEA-KEGG pathway analysis for **(A)** *CS*, **(B)** *CYP1B1*, **(C)** *FLVCR1*, **(D)** *IFIT2*, **(E)** *MAPK14*, and **(F)** *PID1*.

We ran a single-gene version of GSEA-KEGG to investigate the potential function of marker genes. **Figures 4A–F** showed the top six pathways that were enriched for each marker gene. For *CS*, the top three pathways that were enriched in ASTHMA, DNA_REPLICATION, INTESTINAL_IMMUNE_NETWORK_FOR_IGA_PRODUCTION. *CYP1B1* were enriched in CELL_ADHESION_MOLECULES_CAMS, NEUROACTIVE_LIGAND_RECEPTOR_INTERACTION, OLFACTORY_TRANSDUCTION. *FLVCR1* were enriched in ALLOGRAFT_REJECTION, ANTIGEN_PROCESSING_AND_PRESENTATION, GRAFT_VERSUS_HOST_DISEASE. *IFIT2* were enriched in KEGG_ALLOGRAFT_REJECTION, ANTIGEN_PROCESSING_AND_PRESENTATION, GRAFT_VERSUS_HOST_DISEASE. *MAPK14* were enriched in ALLOGRAFT_REJECTION, ANTIGEN_PROCESSING_AND_PRESENTATION, AUTOIMMUNE_THYROID_DISEASE. *PID1* were enriched in ALLOGRAFT_REJECTION, AUTOIMMUNE_THYROID_DISEASE, CELL_CYCLE. After a comprehensive analysis, we found that these genes were enriched in pathways related to immune function and mitochondrial function.

### 3.5 ceRNA networks based on the six diagnostic genes

**Figure 5:**
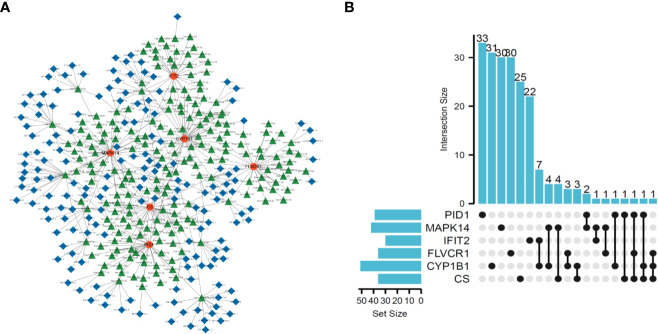
Construction of ceRNA network based on six marker genes. **(A)** ceRNA network based on six marker genes. **(B)** UpSet plot was utilized to present the interaction network of marker genes and miRNAs.

To explore the possible mechanisms involved in the dysregulation of diagnostic genes, we constructed a ceRNA network based on six marker genes using the starBase and miRanda databases. The network included 371 nodes (6 marker genes, 201 miRNAs, and 164 lncRNAs) (**Table S7**), and the specific network is shown in **Figure 5A**. Furthermore, 30 primary miRNAs simultaneously controlled multiple diagnostic genes. Among them, three miRNAs were able to control more than two mRNA, including hsa-miR-630 (controlling *CS*, *FLVCR1*, and *PID1*), hsa-miR-548x-3p (controlling *CS*, *CYP1B1*, and *PID1*), and hsa-miR-590-3p (controlling *CS*, *CYP1B1*, and *FLVCR1*) (**Figure 5B**).

### 3.6 Correlation of six diagnostic genes with the proportion of infiltrating immune cells

**Figure 6:**
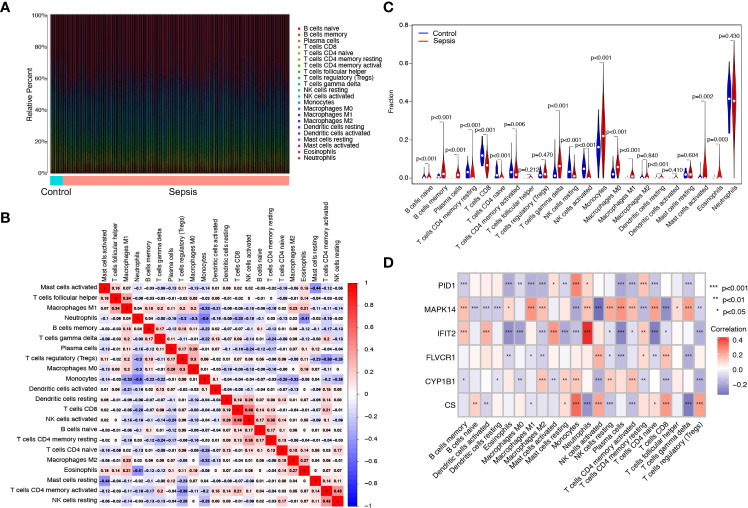
Immune infiltration analysis in septic patients. **(A)** Bar plot illustrating the percentage of 22 different immune cell types found in sepsis samples and normal samples. **(B)** Heatmap displaying the association between 22 different types of immune cells. **(C)** Violin plot showing the ratio differentiation of 22 types of immune cells between normal samples and sepsis samples. **(D)** Heatmap showing the correlation of immune cells with the expression of the six critical genes.

The proportion of infiltrating immune subsets was assessed using the CIBERSORT method, and 22 different immune cell profiles in sepsis samples were created to further demonstrate the association between the six diagnostic genes and the immunological microenvironment. Using the CIBERSORT approach, we investigated the pattern of immune cells. **Figures 6A, B**, respectively, show its makeup in sepsis samples as well as the relationships among immune cells. **Figure 6C** demonstrates that a significant number of immune cells were aberrantly regulated when comparing sepsis samples to normal samples. In addition, Pearson’s correlation analysis revealed that PID1 expression was negatively associated with numerous types of immune cells, such as B cell memory cells, eosinophils, and M0 and M1 macrophages. In addition, PID1 expression was positively associated with resting monocytes and CD4 memory resting T cells (**Figure 6D**). Importantly, we also found that the expression of *CS*, *CYP1B1*, *FLVCR1*, *IFIT2*, and *MAPK14* was associated with many different kinds of immune cells (**Figure 6D**). These data suggest that alterations in the immunological microenvironment of septic patients could be connected to *PID1*, *CS*, *CYP1B1*, *FLVCR1*, *IFIT2*, and *MAPK14*.

### 3.7 Expression and diagnostic value of the diagnostic genes in the external dataset and the cohort dataset

**Figure 7:**
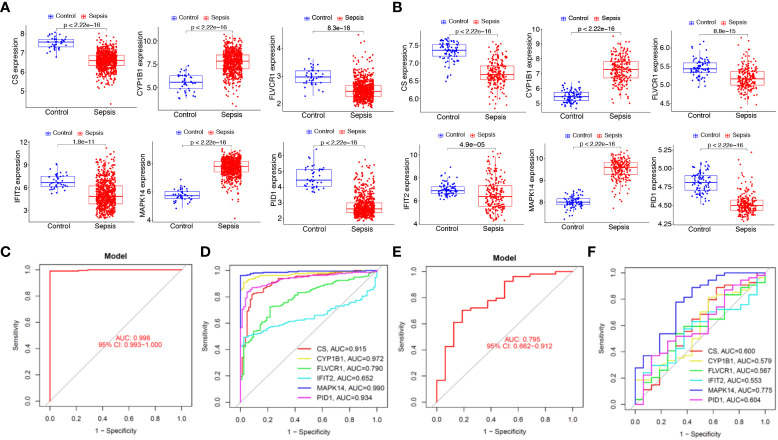
Expression and diagnostic value of the six mitochondrion-related gene signature in the external dataset. Expression of *PID1*, *CS*, *CYP1B1*, *FLVCR1*, *IFIT2*, and *MAPK14* in **(A)** GSE65682 and **(B)** GSE134347 datasets. Diagnostic value of the six mitochondrion-related gene signature was examined in **(C, D)** GSE134347 and **(E, F)** GSE9960 datasets.

We next analyzed the expression of *PID1*, *CS*, *CYP1B1*, *FLVCR1*, *IFIT2*, and *MAPK14* in GSE65682 and GSE134347 datasets. As shown in **Figures 7A, B**, we found that the expression of *MAPK14* and *CYP1B1* was distinctly increased in sepsis samples compared with normal samples, while the expression of *PID1*, *CS*, *FLVCR1*, and *IFIT2* was distinctly decreased in sepsis samples compared with normal samples. Moreover, we analyzed the GSE134347 and GSE9960 datasets using the six-gene diagnostic model, which showed a strong diagnostic value in the GSE134347 dataset with an AUC of 0.998 (**Figures 7C, D**) and in the GSE9960 dataset with an AUC of 0.795 (**Figure 7E, F**).

**Figure 8:**
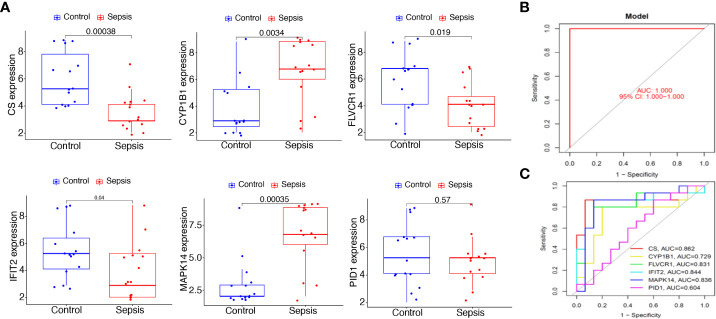
Expression and diagnostic value of the six mitochondrion-related gene signature in the cohort dataset. **(A)** qRT-PCR results for the expression of *PID1*, *CS*, *CYP1B1*, *FLVCR1*, *IFIT2*, and *MAPK14* in our cohort. **(B)** AUC of sepsis samples using ROC analysis. **(C)** ROC analysis for the six critical genes.

To further demonstrate the expression pattern of the diagnostic genes, we collected 15 sepsis samples and 15 normal samples. The results of RT-PCR indicated that *MAPK14* and *CYP1B1* were highly expressed in sepsis samples. In addition, *CS*, *FLVCR1*, and *IFIT2* exhibited lower expression in sepsis samples, which was consistent with the above results (**Figure 8A**). Finally, the new diagnostic model showed good diagnostic value with an AUC of 1.000 (**Figure 8B**). ROC curves were constructed for each of the six genes. As shown in **Figure 8C**, the AUC for *CS*, *CYP1B1*, *FLVCR1*, *IFIT2*, and *MAPK14* was greater than 0.7. Thus, the diagnostic model was further confirmed in our cohort.

### 3.8 Mitochondrial dysfunction in PBMCs from septic patients and LPS-induced CP-M191 cells

**Figure 9:**
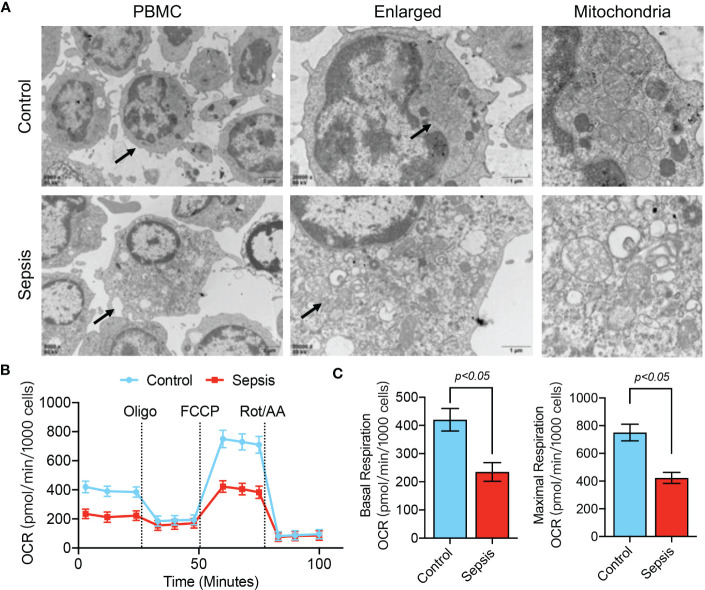
Mitochondrial dysfunction in PBMCs of septic patients. **(A)** TEM analysis of PBMC ultrastructure in healthy controls and septic patients. **(B, C)** Determination of mitochondrial OCR in PBMCs isolated from septic patients and control patients. Baseline OCR and maximal respiratory capacity were recorded.

The above results confirm the value of abnormal mitochondrion-related gene expression in the early diagnosis of sepsis, and we further verified the changes in mitochondrial function in sepsis. We first evaluated mitochondrial morphology. The TEM results showed that the mitochondria in blood PBMCs of the healthy population had good morphology and dense internal cristae structure, while the mitochondria in blood PBMCs of septic patients were severely swollen and showed more vacuolation (**Figure 9A**). Using the Seahorse mitochondrial metabolism analyzer to detect mitochondrial metabolism in blood PBMCs in each patient group, OCR analysis revealed that the maximum respiratory capacity and basal respiratory capacity of mitochondria in blood PBMCs of septic patients were significantly lower (p<0.05) than those of the healthy population (**Figures 9B, C**), suggesting that blood PBMCs of septic patients had severe disorders of mitochondrial metabolism.

**Figure 10:**
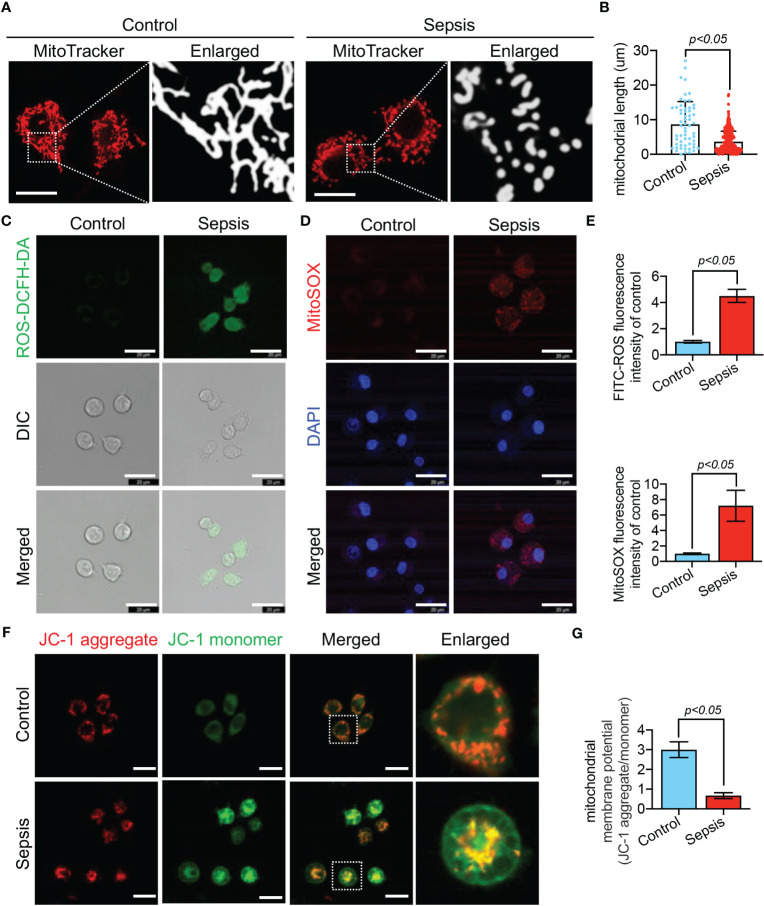
LPS-induced mitochondrial dysfunction in CP-M191 cells. CP-M191 cells were treated *in vitro* with 10 μg/ml LPS for 24 h. **(A, B)** Representative confocal images of CP-M191 cells mitochondrial morphology in each group (bar = 15 μm) and analysis of mitochondrial skeletons using Image J software. **(C)** Representative confocal images of ROS fluorescence intensity in CP-M191 cells (bar = 20 μm). **(D)** Representative images of CP-M191 cells loaded with the mitochondrial superoxide indicator MitoSOX Red to analyze mitochondrial ROS production (bar = 20 μm). **(E)** Statistical analysis of ROS in CP-M191 cells (n = 5). **(F)** Representative confocal images of mitochondrial membrane potential (ΔΨm) of CP-M191 cells, which were labeled with JC-1 monomer (green fluorescent probe) and JC-1 aggregate (red fluorescent probe) (bar = 20 μm). **(G)** Statistical analysis of ΔΨm in CP-M191 cells (n = 5). Data are presented as mean ± standard deviation.

We further observed LPS-stimulated CP-M191 cells in a cellular model wherein we also observed significant changes in mitochondrial morphology (**Figure 10A**), which mainly manifested as mitochondrial fragmentation and excessive mitochondrial division (p<0.05) (**Figure 10B**). The mitochondrial function assay revealed that LPS-stimulated CP-M191 cells had severe ROS accumulation compared with the control group (p<0.05) (**Figure 10C**), which was mainly related to the massive production of mitochondrial ROS (p< 0.05) (**Figures 10D, E**). In addition, confocal microscopy results further revealed that the mitochondrial membrane potential was higher in normal CP-M191 cells, whereas it was significantly reduced after LPS stimulation (p<0.05) (**Figures 10F, G**).

The above results confirm the occurrence of mitochondrial dysfunction in PBMCs of sepsis at both the overall and cellular levels. Therefore, the use of these screened mitochondrion-related biomarkers for sepsis diagnosis is feasible.

## 4 Discussion

The tissue perfusion index, indicators of organ function, inflammatory variables, and hemodynamic indices are among the biomarkers associated with sepsis (36, 37). However, neither the specificity nor the sensitivity of these indicators is sufficient. Therefore, a more accurate diagnosis of sepsis requires the use of biomarkers that have a greater level of both specificity and sensitivity, which are essential for timely and effective treatment and improved prognosis.

Excessive inflammation has been identified as a core determinant of the development of sepsis-related organ injury (38), in addition, recent research has revealed that development of sepsis is associated with alterations in mitochondrial structure and function (39, 40). Sepsis organ dysfunction, such as septic cardiomyopathy, is closely associated with mitochondrial dysfunction (41, 42). Previously, few studies have focused on the value of mitochondria-related genes in the early diagnosis of sepsis. Therefore, the objectives of this study were to search for potential diagnostic biomarkers of sepsis and examine their impact on immune cell infiltration in sepsis.

Through bioinformatics analysis, GEO datasets (GSE65682) was downloaded and 647 mitochondria-related DEGs were identified between septic patients and healthy control. Furthermore, GO and KEGG analyses indicated that these DEGs were mainly associated with mitochondrial transport, energy derivation by oxidation of organic compounds, cellular respiration, mitochondrial gene expression, mitochondrial matrix, mitochondrial inner membrane, electron transfer activity and structural constituent of ribosome. Our findings suggested that mitochondria-related DEGs play an important role in sepsis.

To screen for critical diagnostic MRGs, we performed a LASSO regression algorithm and SVM-RFE methods using 647 mitochondria-related DEGs. Importantly, we identified six critical genes, including *CS*, *CYP1B1*, *FLVCR1*, *IFIT2*, *MAPK14*, and *PID1*, which showed favorable diagnostic value in screening sepsis samples from normal samples. We further developed a diagnostic model based on *CS*, *CYP1B1*, *FLVCR1*, *IFIT2*, *MAPK14*, and *PID1*, and the ROC analysis confirmed the diagnostic value of the novel model with an AUC of 1.000. These results were further confirmed using the GSE9960 and GSE134347 datasets. Finally, we collected 15 sepsis samples and 15 normal samples and performed RT-PCR to examine the expression of *CS*, *CYP1B1*, *FLVCR1*, *IFIT2*, *MAPK14*, and *PID1* in our cohort, which further confirmed our previous findings. Our findings highlight the potential of these 6 MRGs as a novel diagnostic model for sepsis.

Currently, some studies have been conducted to explore the role of the above-mentioned MRGs in the pathophysiology of sepsis. *MAPK14* is a member of the MAP kinase family and is involved in a wide variety of cellular processes such as proliferation, differentiation, and transcription regulation, which can be activated by exposure to many types of cellular stress, among which, they were strongly respond to endotoxin, proinflammatory cytokines, TNF-a, and is a good predictor for sepsis (43). Li et al. found that *MAPK14* is of considerable value in the early diagnosis of sepsis in children (44). Besides, Lu et al. reported that *MAPK14* is up-regulated in sepsis and is closely correlated with responses to hydrocortisone and immunosuppression status and might facilitate personalized therapy (45). Similarly, we found that *MAPK14* is highly expressed in our septic patient cohort and is able to affect immune cell components. Citrate synthase (*CS*) is a key rate-limiting enzyme in many intracellular metabolic pathways and plays a critical role in the tricarboxylic acid cycle by catalyzing oxaloacetate and acetyl coenzyme A. Citrate synthase activity and content depend on the number of mitochondria (46). Moreover, Weiss et al. found that *CS* could be assayed by blood PBMC in response to mitochondrial number/density (47). We found that the *CS* expression of PBMC in septic patients was significantly reduced compared to healthy control, and correspondingly, by *in vitro* experiments, promotion of mitochondrial fragmentation (p<0.05), impaired mitochondrial respiration (p<0.05), decreased mitochondrial membrane potential (p<0.05) and increased ROS generation (p<0.05) were observed in septic patients PBMCs and LPS-stimulated CP-M191 cells. Weiss et al. conducted a study similar to our findings, in which they demonstrated that mitochondrial dysfunction occurs in sepsis, as evidenced by reduced mitochondrial respiratory function and inhibition of *CS* activity (48). Our study confirms that these mitochondria-related gene transcription are altered in sepsis, accompanied by abnormalities in mitochondrial morphology and function.

Sepsis is a severe disorder characterized by an aberrant host response to pathogenic microorganisms and consists of an overwhelming inflammatory response and consequent failure of many organs (49, 50). Although epidemiological data suggest that fatality rates linked with sepsis appear to have decreased, the prevalence of sepsis continues to rise, and the condition is currently considered a substantial burden on health care systems (51). Immunosuppression initiated by sepsis promotes bacterial growth and leads to increased production of immunosuppressive soluble mediators owing to increased apoptosis and immune exhaustion. Sepsis immunosuppression not only prolongs the primary microbial illness, but it also makes the patient more susceptible to opportunistic infections and organ dysfunction, both of which have unfavorable prognoses (52, 53). Numerous protein biomarkers have been tested to differentiate sepsis from normal conditions. Moreover, it has been demonstrated that immune cell infiltration plays a significant role in the progression of sepsis. In this study, Pearson’s correlation analysis revealed that *PID1* expression was negatively associated with many kinds of immune cells, such as B cell memory cells, eosinophils, and M0 and M1 macrophages. In addition, *PID1* expression was positively associated with resting monocytes and CD4 memory resting T cells. This is consistent with previous studies in which *PID1* was able to serve as a biomarker for the assessment of immune status in sepsis and could serve as stratification tools prior to immunostimulatory treatment and to monitor drug efficacy (54). Besides, we found that the sepsis group had higher levels of macrophages M1, T cells gamma delta, etc. compared to the control group, predicting a state of inflammatory activation, consistently, MAPK14 is highly expressed in sepsis and is positively associated with these immune cells, suggesting that MAPK14 may be involved in the development of sepsis through excessive inflammatory activation. Similarly, The expression of CS, CYP1B1, FLVCR1, and IFIT2 were associated with a wide variety of immune cells, which is an important finding. These data suggest that alterations in the immunological microenvironment of septic patients could be connected to *PID1*, *CS*, *CYP1B1*, *FLVCR1*, *IFIT2*, and *MAPK14*.

Our research has certain restrictions. Firstly, although we have analyzed the expression of six MRGs using both clinical samples and datasets, and confirmed the changes in mitochondrial function through *in vitro* sepsis models, additional investigations are required to substantiate the roles of PID1, CS, CYP1B1, FLVCR1, IFIT2, or MAPK14 in the course of sepsis. Secondly, further studies are necessary to elucidate the specific pathways through which these genes influence immune responses in sepsis. As a result, to validate our findings in the future, it is imperative to perform additional experiments both *in vitro* and *in vivo*, along with clinical trials.

## 5 Conclusion

Using bioinformatics techniques, we identified *PID1*, *CS*, *CYP1B1*, *FLVCR1*, *IFIT2*, and *MAPK14* as six MRGs that are essential in the course of sepsis. We developed a diagnostic model based on machine learning that is capable of diagnosing patients as having sepsis by analyzing the expression of a number of genes in the patient’s blood. In addition, these six genes are linked to a variety of immunological components, suggesting that they may play a significant role in the immune microenvironment. Furthermore, by TEM analysis, mitochondrial respiration measurements, mitochondrial potential and mitochondrial ROS production analyses, we observed the occurrence of mitochondrial dysfunction at both the overall and cellular levels, respectively. Additional studies are required to validate the diagnostic potential of this model for sepsis before it can be used in clinical settings.

## Data availability statement

The original contributions presented in the study are included in the article/**Supplementary Material**. Further inquiries can be directed to the corresponding author.

## Ethics statement

The studies involving human participants were reviewed and approved by the Institutional Ethics Committee of The Second Affiliated Hospital of Chongqing Medical University (ID:2022-190). The patients/participants provided their written informed consent to participate in this study.

## Author contributions

SH: Conceptualization, methodology, writing-original draft, software, investigation, funding acquisition. MH: Investigation, software. XX: Formal analysis. XW: Visualization. YS: Validation. WJ: Software. LH: Formal analysis. JG: Supervision, funding acquisition, writing-review & editing. All authors contributed to the article and approved the submitted version.

## Supplementary material

Supplementary Figure 1 Workflow of the study.
