# Supplementary figures and images for: Identification and validation of a novel mitochondrion-related gene signature for diagnosis and immune infiltration in sepsis
Source: Front Immunol. 2023 Jun 15;14:1196306. doi: 10.3389/fimmu.2023.1196306 (PMC10310918; doi:10.3389/fimmu.2023.1196306)

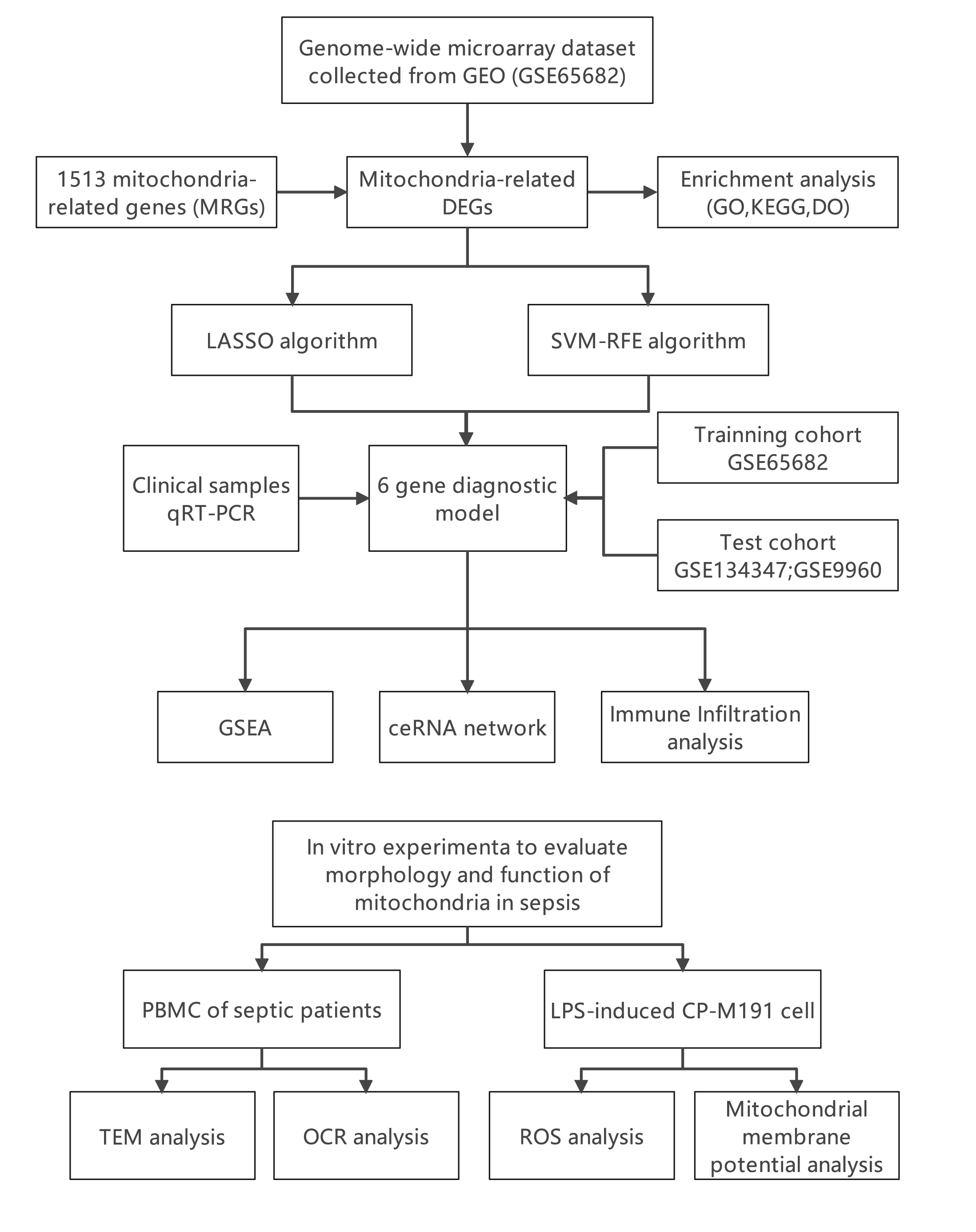

Supplement: Supplementary Figure 1 — Workflow of the study. [file Image_1.tif]
