# Supplementary material for: Identification and validation of a novel mitochondrion-related gene signature for diagnosis and immune infiltration in sepsis
Source: Front Immunol. 2023 Jun 15;14:1196306. doi: 10.3389/fimmu.2023.1196306 (PMC10310918; doi:10.3389/fimmu.2023.1196306)
Supplement: Supplementary file 2 [file Table_1.docx]

Table S1 The names of all Mitochondria-related genes.

Gene names

AADAT

AARS2

AASS

ABAT

ABCB10

ABCB6

ABCB7

ABCB8

ABCD1

ABCE1

ABCG2

ABHD10

ABHD6

ABL1

ACAA2

ACACB

ACAD11

ACAD8

ACAD9

ACADL

ACADM

ACADS

ACADSB

ACADVL

ACAT1

ACBD3

ACLY

ACO2

ACOD1

ACOT11

ACOT13

ACOT2

ACOT7

ACOT9

ACP6

ACSF2

ACSF3

ACSL1

ACSL3

ACSL4

ACSL5

ACSL6

ACSM1

ACSM2A

ACSM2B

ACSM3

ACSM4

ACSM5

ACSM6

ACSS1

ACSS3

ACTN3

ACTR10

ADCK1

ADCY10

ADHFE1

ADPRS

ADSS2

AFG1L

AFG3L2

AGBL4

AGK

AGMAT

AGPAT5

AGTPBP1

AGXT

AGXT2

AIFM1

AIFM2

AIFM3

AK2

AK3

AK4

AKAP1

AKAP10

AKR1B15

AKT1

ALAS1

ALAS2

ALB

ALDH18A1

ALDH1B1

ALDH1L2

ALDH2

ALDH4A1

ALDH5A1

ALDH6A1

ALDH7A1

ALKBH1

ALKBH7

ALOX12

ALPL

AMACR

AMBRA1

AMT

ANK2

ANTKMT

ANXA6

APAF1

APEX1

APEX2

APOO

APOOL

AQP8

ARG2

ARHGAP11B

ARL2

ARL2BP

ARMC1

ARMCX1

ARMCX2

ARMCX3

ARMCX6

ARRB2

ASAH2

ASB9

ATAD1

ATAD3A

ATAD3B

ATCAY

ATF2

ATG12

ATG13

ATG14

ATG2A

ATG2B

ATG3

ATG4D

ATG5

ATG7

ATG9A

ATG9B

ATOX1

ATP12A

ATP13A2

ATP13A3

ATP1B1

ATP1B3

ATP23

ATP2A1

ATP5F1A

ATP5F1B

ATP5F1C

ATP5F1D

ATP5F1E

ATP5F1EP2

ATP5IF1

ATP5MC1

ATP5MC2

ATP5MC3

ATP5ME

ATP5MF

ATP5MG

ATP5MGL

ATP5MJ

ATP5MK

ATP5PB

ATP5PD

ATP5PF

ATP5PO

ATP6AP1

ATP6V1B1

ATP6V1C1

ATP6V1D

ATP6V1E1

ATP6V1F

ATP6V1G1

ATP7A

ATP7B

ATP8A1

ATPAF1

ATPAF2

ATPSCKMT

AUH

AURKAIP1

AVP

BAD

BAK1

BAX

BBC3

BCAP31

BCAT2

BCKDHA

BCKDHB

BCKDK

BCL2

BCL2L1

BCL2L10

BCL2L11

BCL2L13

BCL2L2

BCLAF3

BCO2

BCS1L

BDH1

BECN1

BHLHA15

BID

BIK

BIRC2

BIRC3

BLID

BLOC1S1

BLOC1S2

BMF

BNIP1

BNIP3

BNIP3L

BOK

BOLA1

BOLA3

BOP1

BRAT1

BRAWNIN

BRI3BP

BRINP3

C10orf67

C14orf119

C15orf62

C19orf12

C1orf43

C1QBP

C2orf69

CA5A

CA5B

CABS1

CAMK2A

CAMKK2

CAPRIN2

CARD19

CARS2

CASP2

CASP3

CASP4

CASP6

CASP7

CASP8

CASP8AP2

CASP9

CASQ1

CAVIN1

CBR4

CCAR2

CCDC51

CCDC90B

CCK

CCN6

CCNB1

CDC37

CDK1

CDK5RAP1

CDKN2A

CEBPZOS

CEP89

CFAP410

CFAP91

CFL2

CHCHD1

CHCHD10

CHCHD2

CHCHD2P9

CHCHD3

CHCHD4

CHCHD5

CHCHD6

CHCHD7

CHDH

CHPF

CIAPIN1

CIDEB

CISD1

CISD2

CISD3

CKB

CKMT1A

CKMT1B

CKMT2

CLIC4

CLPB

CLPP

CLPX

CLU

CLUH

CLYBL

CMC1

CMC2

CMC4

CMPK2

CNR1

COA1

COA3

COA4

COA6

COA7

COA8

COASY

COQ10A

COQ10B

COQ2

COQ3

COQ4

COQ5

COQ6

COQ7

COQ8A

COQ8B

COQ9

COX10

COX11

COX14

COX15

COX16

COX17

COX18

COX19

COX20

COX4I1

COX4I2

COX5A

COX5B

COX6A1

COX6A2

COX6B1

COX6B2

COX6C

COX7A1

COX7A2

COX7A2L

COX7A2P2

COX7B

COX7B2

COX7C

COX8A

COX8C

CP

CPOX

CPS1

CPT1A

CPT1B

CPT1C

CPT2

CRAT

CRLS1

CRYZ

CRYZL1

CS

CSKMT

CSNK2A2

CTSK

CTTN

CYB5A

CYB5B

CYB5R3

CYC1

CYCS

CYP11A1

CYP11B1

CYP11B2

CYP1A1

CYP1B1

CYP24A1

CYP27A1

CYP27B1

CYP27C1

CYP2D7

CYP2E1

CYP2U1

CYRIB

D2HGDH

DAP3

DARS2

DBT

DCN

DCTN6

DCTPP1

DCXR

DDAH2

DDIT4

DDX1

DDX21

DDX28

DECR1

DEGS1

DELE1

DEPP1

DERA

DFFA

DFFB

DGLUCY

DGUOK

DHFR

DHFR2

DHODH

DHRS2

DHTKD1

DHX30

DHX32

DHX36

DIABLO

DIP2A

DISC1

DLAT

DLD

DLST

DMAC1

DMAC2

DMAC2L

DMGDH

DMPK

DNA2

DNAJA1

DNAJA3

DNAJC11

DNAJC15

DNAJC19

DNAJC30

DNLZ

DNM1L

DUSP18

DUSP21

DUT

DYNLL1

E2F1

EARS2

ECH1

ECHDC2

ECHDC3

ECHS1

ECI1

ECI2

ECSIT

EFHD1

ELAC2

ENDOG

ENOSF1

ERAL1

ERBB4

ERCC6L2

ETFA

ETFB

ETFBKMT

ETFDH

ETFRF1

ETHE1

ETNPPL

EXD2

EXOG

EYA2

FADS1

FAHD1

FAM162A

FAM210A

FAM210B

FAM72A

FARS2

FASTK

FASTKD1

FASTKD2

FASTKD3

FASTKD5

FATE1

FBXL4

FBXO7

FBXW7

FDX1

FDX2

FDXR

FECH

FEM1A

FEN1

FEZ1

FGR

FH

FHIT

FIBP

FIS1

FKBP4

FKBP8

FLAD1

FLVCR1

FMC1

FMO1

FMO2

FMO5

FOXO3

FOXRED1

FPGS

FTMT

FUNDC1

FUNDC2

FXN

FZD5

FZD9

G0S2

G6PD

GABARAP

GABARAPL1

GABARAPL2

GABARAPL3

GADD45GIP1

GARS1

GATB

GATC

GATD3

GATD3A

GATD3B

GATM

GBA

GCAT

GCDH

GCK

GCKR

GCLM

GCSH

GDAP1

GDF5-AS1

GFER

GFM1

GFM2

GGCT

GHITM

GIMAP5

GIMAP8

GK

GK2

GK3P

GLDC

GLOD4

GLRX2

GLRX5

GLS

GLS2

GLUD1

GLUD2

GLUL

GLYAT

GLYCTK

GOLPH3

GOT2

GPAM

GPAT2

GPD2

GPER1

GPS2

GPX1

GPX4

GRAMD4

GRPEL1

GRPEL2

GRSF1

GSK3A

GSK3B

GSR

GSTO1

GSTP1

GSTZ1

GTPBP3

GUF1

GYG1

GZMA

GZMB

H1-2

H6PD

HADH

HADHA

HADHB

HAGH

HAP1

HARS1

HARS2

HAT1

HAX1

HCCS

HCLS1

HDAC6

HEBP2

HEMK1

HGF

HIBADH

HIBCH

HIF1A

HIF3A

HIGD1A

HIGD2A

HINT2

HIP1R

HK1

HK2

HKDC1

HLCS

HMGCL

HMGCS2

HOGA1

HPDL

HRK

HSCB

HSD17B10

HSD17B8

HSD3B1

HSD3B2

HSDL1

HSP90AA1

HSPA1A

HSPA9

HSPD1

HSPE1

HTD2

HTRA2

HTT

HUWE1

IARS2

IBA57

IDH1

IDH2

IDH3A

IDH3B

IDH3G

IFI27

IFI27L2

IFI6

IFIH1

IFIT2

IFIT3

IGF1

IMMP1L

IMMP2L

IMMT

IRF3

ISCA1

ISCA2

ISCU

IVD

JTB

JUN

KANK2

KARS1

KAT2A

KCNAB2

KDR

KHDC3L

KIF1B

KIF28P

KLK6

KMO

L2HGDH

LACTB

LACTB2

LARS2

LDHB

LDHD

LETM1

LETM2

LETMD1

LIAS

LIG3

LIPT1

LIPT2

LONP1

LRPPRC

LRRK2

LYN

LYRM4

LYRM7

MAIP1

MALSU1

MAOA

MAOB

MAP1B

MAP1LC3A

MAP1LC3B

MAP1LC3B2

MAP1LC3C

MAP1S

MAPK10

MAPK12

MAPK14

MAPK8

MAPK8IP1

MAPT

MARCHF5

MARK1

MARK2

MARS2

MAVS

MCAT

MCCC1

MCCC2

MCCD1

MCEE

MCL1

MCU

MCUB

MCUR1

MDH1

MDH2

ME1

ME2

ME3

MECR

MEF2A

METAP1D

METTL13

METTL15

METTL17

METTL4

METTL9

MFF

MFI

MFN1

MFN2

MGARP

MGME1

MGST1

MICOS10

MICOS13

MICU1

MICU2

MICU3

MIEF1

MIEF2

MIGA1

MIGA2

MIPEP

MIR17

MIR210

MIR29A

MIR29B1

MIR29C

MLLT11

MLXIP

MLXIPL

MLYCD

MMAA

MMAB

MMADHC

MMP2

MMP9

MMUT

MOAP1

MPC1

MPC2

MPG

MPST

MPV17

MPV17L

MPV17L2

MRM1

MRM2

MRM3

MRPL1

MRPL10

MRPL11

MRPL12

MRPL13

MRPL14

MRPL15

MRPL16

MRPL17

MRPL18

MRPL19

MRPL2

MRPL20

MRPL21

MRPL22

MRPL23

MRPL24

MRPL27

MRPL28

MRPL3

MRPL30

MRPL32

MRPL33

MRPL34

MRPL35

MRPL36

MRPL37

MRPL38

MRPL39

MRPL4

MRPL40

MRPL41

MRPL42

MRPL43

MRPL44

MRPL45

MRPL46

MRPL47

MRPL48

MRPL49

MRPL50

MRPL51

MRPL52

MRPL53

MRPL54

MRPL55

MRPL57

MRPL58

MRPL9

MRPS10

MRPS11

MRPS12

MRPS14

MRPS15

MRPS16

MRPS17

MRPS18A

MRPS18B

MRPS18C

MRPS2

MRPS21

MRPS22

MRPS23

MRPS24

MRPS25

MRPS26

MRPS27

MRPS28

MRPS30

MRPS31

MRPS33

MRPS34

MRPS35

MRPS36

MRPS5

MRPS6

MRPS7

MRPS9

MRRF

MRS2

MSH2

MSRA

MSRB2

MSRB3

MSTO1

MTARC1

MTARC2

MT-ATP6

MT-ATP8

MTCH1

MTCH2

MT-CO1

MT-CO2

MTCO2P12

MT-CO3

MT-CYB

MTERF1

MTERF2

MTERF3

MTERF4

MTFMT

MTFP1

MTFR1

MTFR2

MTG1

MTG2

MTHFD1L

MTHFD2

MTHFD2L

MTIF2

MTIF3

MTLN

MTM1

MT-ND1

MT-ND2

MT-ND3

MT-ND4

MT-ND4L

MT-ND5

MT-ND6

MTO1

MTOR

MTPAP

MTRES1

MTRF1

MTRF1L

MTRFR

MT-RNR1

MT-RNR2

MTRNR2L5

MTRR

MT-TF

MT-TH

MT-TL1

MT-TL2

MT-TN

MT-TQ

MT-TS2

MT-TW

MTUS1

MTX1

MTX2

MTX3

MUC1

MUL1

MUTYH

MYCBP

MYG1

MYH7

MYO19

MYOC

MYOG

NADK

NADK2

NAGS

NAIF1

NARS2

NAT8L

NAXD

NAXE

NDUFA1

NDUFA10

NDUFA11

NDUFA12

NDUFA13

NDUFA2

NDUFA3

NDUFA4

NDUFA5

NDUFA6

NDUFA7

NDUFA8

NDUFA9

NDUFAB1

NDUFAF1

NDUFAF2

NDUFAF3

NDUFAF4

NDUFAF5

NDUFAF6

NDUFAF7

NDUFAF8

NDUFB1

NDUFB10

NDUFB11

NDUFB2

NDUFB3

NDUFB4

NDUFB5

NDUFB6

NDUFB7

NDUFB8

NDUFB9

NDUFC1

NDUFC2

NDUFC2-KCTD14

NDUFS1

NDUFS2

NDUFS3

NDUFS4

NDUFS5

NDUFS6

NDUFS7

NDUFS8

NDUFV1

NDUFV2

NDUFV3

NECTIN2

NEFL

NENF

NEU4

NFS1

NFU1

NGB

NGRN

NIPSNAP1

NIPSNAP2

NIT1

NLN

NLRP5

NLRX1

NME4

NMNAT3

NMT1

NNT

NOA1

NOCT

NOD2

NOL3

NOX1

NQO1

NR3C1

NR4A1

NRP1

NSUN2

NSUN3

NSUN4

NT5M

NTHL1

NUBPL

NUDT1

NUDT12

NUDT13

NUDT17

NUDT6

NUDT8

NUDT9

NUPR1

OAS1

OAT

OGDH

OGDHL

OGG1

OGT

OLFM4

OMA1

OPA1

OPA3

OPTN

OSGEPL1

OTC

OXA1L

OXCT1

OXCT2

OXR1

OXSM

PAAT

PABPC5

PACS2

PAGE4

PAK5

PAM16

PANK2

PARG

PARK7

PARL

PARP1

PARS2

PC

PCCA

PCCB

PCK2

PDCD5

PDE12

PDE2A

PDF

PDHA1

PDHA2

PDHB

PDHX

PDK1

PDK2

PDK3

PDK4

PDP1

PDP2

PDPR

PDSS1

PDSS2

PDZD8

PEMT

PET100

PET117

PFDN2

PFDN4

PGAM1

PGAM5

PGD

PGLS

PGM2

PGR

PGRMC1

PGS1

PHB

PHB2

PHYKPL

PI4K2A

PI4KB

PID1

PIF1

PIGBOS1

PIN4

PINK1

PISD

PITRM1

PLA2G2A

PLA2G4B

PLA2G4C

PLA2G4F

PLA2G6

PLAAT3

PLAUR

PLD6

PLEKHN1

PLIN5

PLN

PLS3

PLSCR3

PMAIP1

PMPCA

PMPCB

PNKD

PNPLA4

PNPLA8

PNPT1

POLDIP2

POLG

POLG2

POLRMT

PPA2

PPARGC1A

PPARGC1B

PPID

PPIF

PPM1K

PPOX

PPP1CC

PPP1R13B

PPP1R15A

PPP2CB

PPP2R2B

PPP3CC

PPP3R1

PPP6C

PPTC7

PRDX3

PRDX5

PRELID1

PRELID3A

PRICKLE3

PRIMPOL

PRKAA1

PRKACA

PRKCA

PRKCD

PRKG1

PRKN

PRNP

PRODH

PRORP

PSMD10

PTCD1

PTCD2

PTCD3

PTPMT1

PTPN1

PTRH2

PUS1

PUS10

PYCARD

PYCR1

PYCR2

PYROXD2

PYURF

QRSL1

QTRT1

QTRT2

RAB11FIP5

RAB32

RAB40AL

RAB5IF

RACK1

RAD51

RAD51C

RAF1

RALA

RALBP1

RAP1GDS1

RARS2

RB1CC1

RBFA

RBKS

RCC1L

RDH13

REEP1

REXO2

RGS2

RHBDD1

RHOT1

RHOT2

RIDA

RMDN3

RMND1

RNASEL

RNASET2

RNF144B

RNF185

RNF41

RNF5

ROMO1

RPE

RPEL1

RPIA

RPS3

RPS6KB1

RPUSD3

RPUSD4

RSAD1

RSAD2

RTL10

RTN4IP1

RXRA

S100A1

SAMM50

SARDH

SARM1

SARS2

SCO1

SCO2

SCP2

SDHA

SDHAF1

SDHAF2

SDHAF3

SDHAF4

SDHB

SDHC

SDHD

SEC61G

SECISBP2

SELENOO

SEPTIN4

SERAC1

SERPINB5

SFN

SFXN1

SFXN2

SFXN3

SFXN4

SFXN5

SGK1

SH3BP5

SH3GLB1

SHC1

SHMT2

SHPK

SIAH3

SIRT1

SIRT2

SIRT3

SIRT4

SIRT5

SIRT6

SIRT7

SIVA1

SLC11A2

SLC16A3

SLC16A5

SLC22A14

SLC25A1

SLC25A10

SLC25A11

SLC25A12

SLC25A13

SLC25A14

SLC25A15

SLC25A16

SLC25A17

SLC25A18

SLC25A19

SLC25A2

SLC25A20

SLC25A21

SLC25A22

SLC25A23

SLC25A24

SLC25A25

SLC25A26

SLC25A27

SLC25A28

SLC25A29

SLC25A3

SLC25A30

SLC25A31

SLC25A32

SLC25A33

SLC25A34

SLC25A35

SLC25A36

SLC25A37

SLC25A38

SLC25A39

SLC25A4

SLC25A40

SLC25A41

SLC25A42

SLC25A43

SLC25A44

SLC25A45

SLC25A46

SLC25A47

SLC25A48

SLC25A5

SLC25A51

SLC25A52

SLC25A53

SLC25A6

SLC27A3

SLC35F6

SLC40A1

SLC44A1

SLC4A5

SLC8A3

SLC8B1

SLC9A2

SLC9A5

SLC9B2

SLIRP

SMCP

SMDT1

SMIM20

SNCA

SNN

SOD1

SOD2

SORD

SOX10

SPAST

SPATA18

SPATA19

SPATA5

SPG7

SPHK2

SPNS1

SQOR

SQSTM1

SRC

SREBF1

SREBF2

SSBP1

STAP1

STAR

STARD13

STARD7

STING1

STK11

STMP1

STOML2

STPG1

STYXL1

SUCLA2

SUCLG1

SUCLG2

SUGCT

SUOX

SUPV3L1

SURF1

SYBU

SYNE2

SYNJ2BP

TACO1

TAFAZZIN

TALDO1

TAMM41

TARS2

TAZ

TBRG4

TCAIM

TCHP

TDH

TDRKH

TEFM

TFAM

TFB1M

TFB2M

TFDP1

TFDP2

TFRC

TGM2

THEM4

THEM5

THG1L

TICAM1

TIGAR

TIMM10

TIMM10B

TIMM13

TIMM17A

TIMM17B

TIMM21

TIMM22

TIMM23

TIMM23B

TIMM29

TIMM44

TIMM50

TIMM8A

TIMM8B

TIMM9

TIMMDC1

TK2

TKT

TLE6

TMEM102

TMEM11

TMEM126A

TMEM126B

TMEM135

TMEM14A

TMEM14C

TMEM177

TMEM65

TMEM70

TMEM8B

TMLHE

TMX2

TNFSF10

TOMM20

TOMM20L

TOMM22

TOMM34

TOMM40

TOMM40L

TOMM5

TOMM6

TOMM7

TOMM70

TOP1MT

TOP3A

TP53

TP53AIP1

TP53BP2

TP53I3

TP63

TP73

TRAF3

TRAF3IP3

TRAK1

TRAK2

TRAP1

TRIAP1

TRIM14

TRIM31

TRIM39

TRIM45

TRIT1

TRMT10A

TRMT10B

TRMT10C

TRMT5

TRMT61B

TRMU

TRNT1

TRUB2

TSC2

TSFM

TSPO

TSPOAP1

TST

TTC19

TUFM

TWNK

TXN

TXN2

TXNRD2

TYMP

TYMS

UBA1

UBB

UBIAD1

UCP1

UCP2

UCP3

UNG

UQCC1

UQCC2

UQCC3

UQCR10

UQCR11

UQCRB

UQCRC1

UQCRC2

UQCRFS1

UQCRFS1P1

UQCRH

UQCRHL

UQCRQ

URI1

USP15

USP30

USP36

UXT

VAMP1

VARS2

VAT1

VCP

VDAC1

VDAC2

VDAC3

VPS13A

VPS13C

VPS13D

VPS35

VRK2

VWA8

WARS2

WASF1

WDR26

WDR35

WDR45

WDR45B

WDR81

WDTC1

WIPI1

WIPI2

WWOX

XAF1

XIAP

XPNPEP3

XRCC3

YARS2

YME1L1

YRDC

YWHAB

YWHAE

YWHAG

YWHAH

YWHAQ

YWHAZ

ZBED3

ZDHHC6

ZDHHC8

ZFYVE1

ZNF205
