# Supplementary material for: Identification and validation of a novel mitochondrion-related gene signature for diagnosis and immune infiltration in sepsis
Source: Front Immunol. 2023 Jun 15;14:1196306. doi: 10.3389/fimmu.2023.1196306 (PMC10310918; doi:10.3389/fimmu.2023.1196306)
Supplement: Supplementary file 4 [file Table_3.docx]

| Gene | Analysis | Forward Primer (5' to 3') | Reverse Primer (5' to 3') |
| --- | --- | --- | --- |
| PID1 | mRNA | GGGCAAAGTCTCCACCACTG | GTCGAGATGATGGAGCCAAACT |
| CS | mRNA | AGTTGGCAAAGATGTGTCAGATGA | GCACAATCTTGTACAGCTGAGCA |
| CYP1B1 | mRNA | CGAATTCGAGCAGCTCAACC | CGGTACGTTCTCCAAATCCAG |
| FLVCR1 | mRNA | CCATGTTTTATGGAACATCAGCTGT | GGGACTGTCTTGAAGAGCTGCTT |
| IFIT2 | mRNA | CATTTCACCTGGAACTTGATGGAG | TTACGTAAGCATTCCAGGGCTG |
| MAPK14 | mRNA | GTTCTACCGGCAGGAGCTGAA | GACTGAAATGGTCTGGAGAGCTTCT |
| β-actin | mRNA | AATCGTGCGTGACATTAAGGAG | ACGTGTTGGCGTAACAGGTCTT |

Table S3. Primer sets used for qRT-PCR analysis.
